# Molecular chaperoning helps safeguarding mitochondrial integrity and motor functions in the Sahara silver ant *Cataglyphis bombycina*

**DOI:** 10.1038/s41598-018-27628-2

**Published:** 2018-06-15

**Authors:** Quentin Willot, Patrick Mardulyn, Matthieu Defrance, Cyril Gueydan, Serge Aron

**Affiliations:** 1 Evolutionary Biology and Ecology, Université Libre de Bruxelles, CP 160/12, Av. F.D. Roosevelt, 50, Brussels, 1050 Belgium; 2 Interuniversity Institute of Bioinformatics in Brussels, Université Libre de Bruxelles, Boulevard du Triomphe, Brussels, 1050 Belgium; 3 Molecular Biology of the Gene, Université Libre de Bruxelles, Rue des Profs. Jeener et Brachet, 12, Gosselies, 6041 Belgium

## Abstract

The Sahara silver ant *Cataglyphis bombycina* is one of the world’s most thermotolerant animals. Workers forage for heat-stricken arthropods during the hottest part of the day, when temperatures exceed 50 °C. However, the physiological adaptations needed to cope with such harsh conditions remain poorly studied in this desert species. Using transcriptomics, we screened for the most heat-responsive transcripts of *C. bombycina* with aim to better characterize the molecular mechanisms involved with macromolecular stability and cell survival to heat-stress. We identified 67 strongly and consistently expressed transcripts, and we show evidences of both evolutionary selection and specific heat-induction of mitochondrial-related molecular chaperones that have not been documented in *Formicidae* so far. This indicates clear focus of the silver ant’s heat-shock response in preserving mitochondrial integrity and energy production. The joined induction of small heat-shock proteins likely depicts the higher requirement of this insect for proper motor function in response to extreme burst of heat-stresses. We discuss how those physiological adaptations may effectively help workers resist and survive the scorching heat and burning ground of the midday Sahara Desert.

## Introduction

Temperature plays a key role in protein homeostasis^1^. Most peptides are stable within a narrow thermal range, and increases or decreases in temperature can cause them to unfold and form denatured aggregates^1,2^. Such sensitivity likely led to the early evolutionary appearance of the heat-shock response (HSR)^3,4^. One of the HSR’s main functions is to increase macromolecular stability, which helps organisms cope efficiently with thermal shifts, as well as oxidative stress, heavy metal contamination, or exposure to toxins^5,6^. A major component of the HSR is the transcriptional response, which is controlled by several factors including the evolutionarily conserved transcriptional activator heat-shock factor 1 (HSF1)^7^. HSF1 trimerizes upon heat shock and binds to consensus heat-shock elements (HSEs) localized on promotor regions of target genes^8^. This response, triggered by the presence of unfolded proteins, leads to the fast and transient transcription of target genes, such as heat-shock proteins (*hsps*). Hsps are a large family of molecular chaperones. Their upregulation and accumulation are associated with thermal hardiness^9,10^. Therefore, the HSR in general, and especially Hsps production, play a central role in allowing cells to survive deleterious conditions.

Using transcriptomics, we examined the predominant molecular level processes involved with macromolecular stability and cell survival in the Sahara silver ant, *Cataglyphis bombycina*, focusing on heat-shock proteins. This species forages during the hottest part of the day, scavenging the bodies of less tolerant, heat-stricken arthropods^11^. Workers thus experience harsh conditions: air and ground temperatures can reach as high as 50 °C and 70 °C, respectively^11^. The silver ant manages this feat thanks to its remarkable ability to survive elevated body temperatures (CT_max_ = 53.6 °C)^12^. Previous studies have shown that foragers exhibit high constitutive levels of heat-shock cognate 70 (Hsc70)^13,14^, suggesting that the ants can handle sudden heat exposure without needing to acclimate. However, a deeper understanding of mechanisms involved with the ability of cells to survive heat stress while maintaining high metabolic requirements associated with foraging is still lacking in the silver ant. Our aim in this study was to gain a better understanding of the molecular response underlying *C. bombycina*’s ability to survive such elevated body temperatures for short periods of time.

## Results

### Identification of heat-induced transcripts

We performed a differential gene expression analysis between 4 groups of heat-stressed (4 h; 45 °C) and 4 groups of control workers (25 °C, 4 h). A total of 301,363 putative transcripts (including isoforms) were identified. After removing transcripts with low expression levels, 40,988 transcripts remained. Of these, 533 displayed a significant regulation in response to heat stress (FDR < 0.05; Fig. S1) and were qualified as differentially expressed sequences (DESs). Expression was downregulated for 147 DESs and upregulated for 386 DESs. Most displayed a high degree of fold change (FC) between the two conditions; there was also marked variance in transcription within the heat-stress treatment.

### Similarity annotation

When the 533 DESs were queried against the NCBI nr protein database, 466 sequences (87%) with a high degree of homology were retrieved. Annotation was reliable, as most hits had e-values of less than 1e^−180^ (Fig. S2).

To better characterize the number and function of the genes involved in the HSR, we further filtered the transcripts. A given transcript was retained only if (*i*) mean FC was greater than 2 between heat-stressed and control ants; (*ii*) FDR was less than 0.05; and (*iii*) the relative standard deviation (RSD) of expression between biological replicates was less than 0.4. We were thus left with a total of 67 strongly and consistently expressed transcripts. For this transcript subset, the most represented taxa for the best hit of each match were mainly other ant species, particularly those in the subfamily *Formicinae*, such as *Camponotus floridanus*, a result that reflects *C. bombycina*’s phylogenetic history (Fig. S3).

### Gene ontology annotation

Based on sequence homology, GO terms could be assigned to 393 (73%) of the 533 DESs. The transcripts were distributed across the three GO-classification domains: cellular component (GO levels 5–8), biological process (GO levels 4–8), and molecular function (GO levels 3–5) (Fig. S4).

**Figure 1:**
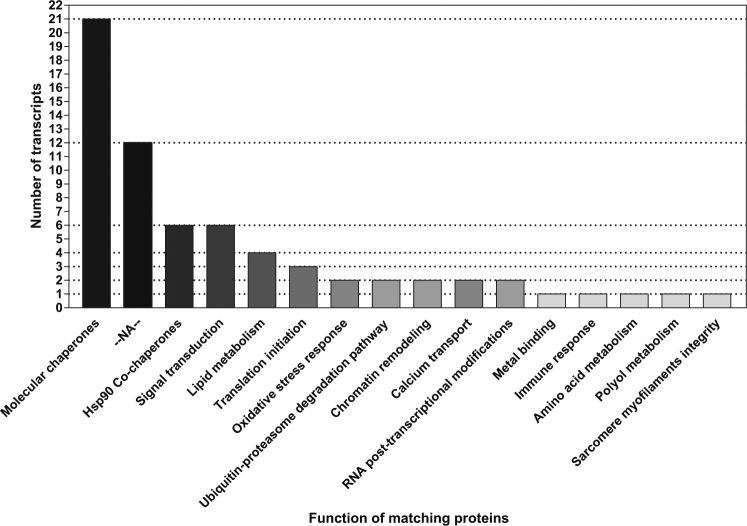
Functional classification of the 67 transcripts with strong and consistent heat-induced expression. Twenty-one transcripts matched up with heat-shock proteins and molecular co-chaperones (31%). Smaller groups of transcripts (<10% each) matched up with proteins with roles ranging from cell-signal transduction to sarcomere myofilament organization.

**Figure 2:**
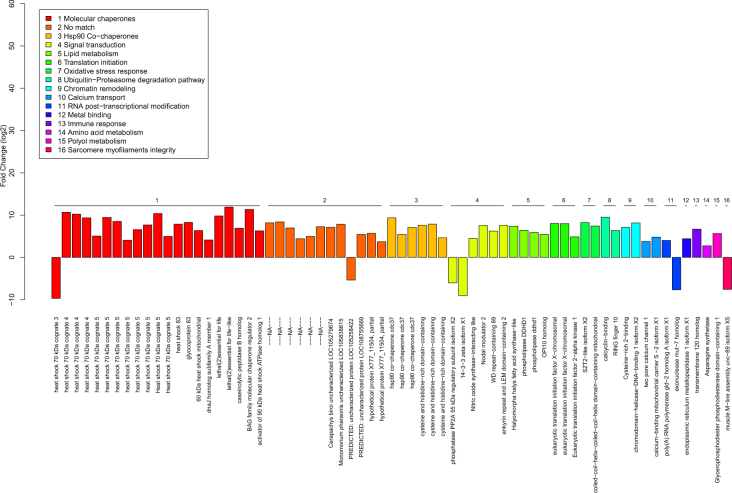
Average fold changes of the 67 strongly and consistently expressed transcripts induced by heat-stress in *C. bombycina* workers. The numeric colour-codes correspond to the following functional classes of proteins: 1: Molecular Chaperones; 2: No match; 3: Hsp90 co-chaperones; 4: Cell signal transduction proteins; 5: Lipid metabolism enzymes; 6: Translation initiation factors; 7: Oxidative stress response proteins; 8: Proteins involved with the ubiquitin-proteasome degradation pathway; 9:Chromatin remodeling proteins; 10: Calcium transport proteins; 11: RNA-modifying proteins; 12: Metal binding proteins; 13: Proteins involved in the immune response; 14: Proteins involved in the amino-acid metabolism; 15: proteins involved in the polyol metabolism; 16: proteins involved in the sarcomere organization.

Fifty-five of the 67 transcripts matched up with various types of proteins, most commonly Hsps and molecular co-chaperones (21 transcripts; 32%; Fig. 1). Notably, smaller groups of transcripts (less than 10% each) matched up with proteins with roles ranging from HSP90 co-chaperones involved in cell division to chromatin remodelling and sarcomere myofilament organization. Results, including the FC in expression, are depicted in Fig. 2. Complete heat-map of heat-induced transcripts is displayed in Fig. S5.

### Molecular chaperones involved in the heat-shock response

Of the 533 DESs, 36 were associated with either molecular chaperones or with co-chaperones involved in protein folding. Of the 67 strongly and consistently expressed transcripts, 21 (31%) of such transcripts remained, indicating that chaperones and co-chaperones were a major part of the HSR (Fig. 1). We found members among the 5 major conserved families of Hsps (Table 1): one transcript matched up with the caseinolytic peptidase B homolog protein (ClpB) which belongs to the Hsp100 family, two with proteins in the Hsp90 family (namely the Gp93 protein and Hsp83), twelve with Hsp70 proteins, one with the Hsp60 mitochondrial molecular chaperone and two with the protein protein Efl21l encoded by *lethal(2)essential for life (l(2)efl)*, which is a member of the Hsp20 family. Among molecular co-chaperones, one transcript matched up with DnaJ homolog subfamily A member 1 (DnajA1) which belongs to the Hsp40 family, one with the BCL-2-associated athanogene protein 2 (BAG-2), and one transcript was associated with the activator of 90-kDa heat-shock protein ATPase homolog 1 (AHA1).

Under conditions of heat stress, it appeared that the transcripts associated with Hsc70-4, protein Efl21l, and BAG-2 were the most upregulated (>10 FC). The only transcript strongly downregulated by heat stress (>8 FC) was associated with Hsc70-3 (Fig. 2).

**Table 1:** Summary of the molecular chaperones and folding co-chaperones found among the 67 consistently heat-induced transcripts of the ant *C*.

Protein family	Protein	Number of associated transcripts	Significant selection along the *Cataglyphis* lineage	*Cataglyphis*-specific heat induction
**Molecular chaperones**				
Hsp100	ClpB	1	−	N.A
Hsp90	Hsp83	1	−	−
	Gp93	1	−	N.A
Hsp70	Hsc70-5	8	+	+
	Hsc70-4h1	2	−	−
	Hsc70-4h2	1	+	−
	Hsc70-3	1	−	N.A
Hsp60	Hsp60 mitochondrial	1	−	+
Hsp20	Efl21	2	−	+
**Co-chaperones**				
Hsp40	DnajA1	1	−	−
BAG proteins	BAG2	1	−	N.A
AHA1	AHA1	1	−	N.A

*bombycina*, queried against the NCBI non-redundant protein database (arthropod records only) using BLASTX (<10e^−5^), complemented by their detected significant selection along the *Cataglyphis* lineage, and their detected specific heat induction as compared to other ants (N.A: not applicable; heat-induction of the gene has not been tested in other ant genera).

### KEGG annotation of the transcripts

For the 533 DESs, the main KEGG pathways involved ribosomes (71; 13%), metabolic processes (54; 10%), secondary metabolite biosynthesis (22; 4%), protein processing in the endoplasmic reticulum (ER) (18; 3%) (Fig. S6), and PI3K-Akt signaling (14; 3%) (Fig. S7) which is part of cell cycle regulation and apoptosis. For the transcripts associated with metabolic processes, there was clear enrichment in the non-oxidative pentose phosphate pathway and the lipid metabolic pathway.

For the 67 strongly and consistently expressed transcripts, the two top KEGG pathways were protein processing in the ER (8; 11%) and PI3K-Akt signaling (4; 6%).

### Detection of patterns of selection

All *d*_N_/*d*_S_ ratios calculated for 32 coding sequences (CDS) between *C. bombycina* and the closely related ant *Camponotus floridanus* were largely below 1, with values ranging from 0.01 to 0.29 (Tables S1 and S2). This shows that all analyzed CDS are predominantly under negative selection, which is typical for a functional protein coding gene since non-synonymous mutations are more likely to generate a disadvantageous allele than an advantageous one.

However, even for proteins characterized by a *d*_N_/*d*_S_ ratio below 1, a signal of positive selection can be detected by highlighting a specific lineage for which this ratio is significantly higher than the background ratio estimated for all other lineages. From the four transcripts for which we have conducted this test (Table S2), a signal of positive selection was detected for the CDS sequences of *hsc70-4 h2* (*d*_N_/*d*_S_ ratio 3.8 times larger along the branch leading to *Cataglyphis*; *p*-value < 0.01) and of *hsc70-5* (*d*_N_/*d*_S_ ratio 2.3 times larger along the branch leading to *Cataglyphis* and along the branches inside the clade *Cataglyphis; p*-value < 0.05; Table 1). These results provide some evidence that positive selection occurred more frequently for these two genes in *Cataglyphis*, compared to what happened in other ant species, *i.e*. that a higher proportion of non-synonymous mutations were favored by selection.

## Discussion

So far, understanding the molecular level processes related to heat tolerance in eusocial Hymenoptera (ants, bees, wasps) has been limited to phylogeny and induction patterns of some Hsps across species and genera^13–17^. Our study investigates further gene expression patterns in response to heat stress using DGE analysis. It shows that of the 67 strongly and consistently expressed transcripts, 21 were linked to proteins that exercise either direct or indirect molecular chaperone folding activity (31%). This protein class was therefore the most responsive to heat stress. As compared, only two transcripts matched up with proteins in the ubiquitin-proteasome pathway (RNF10; a member of the E3 ubiquitin ligase family and CACYBP; which may regulate calcium-dependent ubiquitination and degradation of target peptides)^18^. Higher eukaryotes tend to rely more on refolding to clear misfolded proteins while bacteria tend to exploit degradation pathways^19,20^. Accordingly, our results suggest that *C. bombycina* invests more in its protein-refolding machinery in response to heat stress than in maintaining proteostasis by increased turnover of damaged peptides.

Molecular chaperones are essential for protein synthesis, folding, and translocation under both normal and stressful conditions^9^. Among them, the five major conserved families of Hsps were represented (in order of prominence): Hsp70s, Hsp90s, Hsp60s, Hsp100s and small Hsps (Table 1). Here, we document heat-inducibility for the first time among ants for several of them: *hsc70-5*, *hsp-60 mitochondrial*, as well as the small heat-shock protein *l(2)efl*. Analysis of *C. bombycina* molecular chaperones associated transcripts showed that the Hsp70 family was the most prominent—it was associated with 12 transcripts. Among this family, *hsc70-4* and *hsc70-3* were the only two transcripts already expressed at 25 °C. While similar to other ants, *hsc70-4* exhibited among the greatest induction in expression^14–16^, *hsc70-3* was the only molecular chaperone to show down-regulation in response to heat-stress. The effects of such down-regulation remain so far unclear with regards to molecular chaperoning and stress-tolerance and would deserve further investigation.

We performed tests of positive selection on the coding sequences belonging to the 67 consistently and strongly expressed transcripts in response to heat stress from multiple ant species (see Tables S1 and S2). They indicated a significant increase of positive selection for *hsc70-4 h2* and *hsc70-5* in the *Cataglyphis* lineage. All *Cataglyphis* species are thermal scavengers known to forage at the warmest hours of the day^21^. Consequently, specific evolution of (at least some) molecular chaperones likely occurred in the genus to provide stronger support for macromolecular stability. Remarkably, we found eight isoforms of *hsc70-5*. This finding makes *hsc70-5* isoforms the most numerous *hsps* whose expression is upregulated in response to heat stress in this species. Previous qPCR experiments confirmed heat-inducible expression of *hsc70-5* in *Cataglyphis*^14^. In contrast, the gene *hsc70-5* was not shown to be heat-inducible in the wood ant *Aphaenogaster picea* nor in the harvester ant *Pogonomyrmex barbatus*^15^. Canonical forms of HSEs in the promoter region of *hsc70-5* are variable and lacking in many ants^15^, which could partly explain the observed divergences in induction patterns among genera. Such confirmed, evolutionary selected and highly diversified use of *hsc70-5* in response to heat-stress seems thus so far to be restricted, among ants, to *Cataglyphis*. Importantly, it has been documented that *hsc70-5* plays a critical role in maintaining mitochondria morphology and cellular homeostasis: knockdown of *hsc70-5* in *Drosophila melanogaster* results in severe mitochondria dysfunction as well as reduced viability, locomotion impairment, body posture defects, and reduced ATP levels^22,23^. Consistently, our results in the silver ant show a significant heat-inducibility of *hsp60* (coding for the 60 kDa heat shock protein mitochondrial) that was not reported in other ant taxa investigated so far either^15^. In addition, several transcripts were linked to two Hsp70 cofactors and potential complex partners: Hsp40 (DnaJ), BAG2, and the Hsp100 ClpB homolog (Table 1). While both Hsp40 and BAG2 greatly enhance the Hsp70 folding function^24,25^, ClpB forms a complex with Hsp70/Hsp40 proteins that disaggregates and solubilizes denatured protein aggregates in an ATP-dependent manner^26^. This indicates that Hsp70 family folding activity is critical to cope with stresses in *Cataglyphis*. Altogether, these results confirm the importance of the folding activity of the hsp70 family to face adverse heat-shocks. Furthermore, whether directly via the HSF1 pathway or indirectly by heat-induced oxidative damages to the mitochondria^27^, the joined induction of both *hsc70-5* and *hsp60* supports evidence of a major focus of the silver ant in safeguarding mitochondria integrity and energy production in response to higher temperatures.

The small heat shock proteins (sHsps) family was represented by two transcripts that matched up with the protein Efl21 in *Drosophila* (encoded by *l(2)efl*), which is the ortholog of the Alpha-crystallin B chain in vertebrates (encoded by *CRYAB*)^28^. sHsps bind to and hold unfolded proteins in specific conformations, allowing folding machinery composed of other chaperones to operate^29^. In *D. melanogaster*, Efl21 stabilizes intermediate filament proteins and prevents them from aggregating under deleterious conditions, thus ensuring the structural integrity of the cytoskeleton, organelle morphology and the myofilaments^28^. Ants have three to six copies of *l(2)efl* that lack the putative HSEs^15^ and accordingly, gene expression was not heat inducible in the two species tested so far, *A. picea* and *P. barbatus*. In contrast, our data indicate that expression of the two *l(2)efl* transcripts was strongly heat inducible in *C. bombycina*. Furthermore, three strongly expressed transcripts were each associated with specific proteins involved in muscle structure and function: muscle M-line assembly protein unc-89, two pore calcium channel protein 1 (TPC1), and nitric oxide synthase interacting protein (NOSIP). These three proteins are essential for Ca^2+^ signaling during muscle contraction, and unc-89 is also involved in the assembly and organization of sarcomere^30–32^. This suggests that the HSR in *C. bombycina* also at least partially involves safeguarding muscle tissue organization. The Sahara silver ant is one of world’s fastest running insects: its speed helps escape potential heat damage inflicted by ground temperatures of up to 70 °C^11,21^. Loss of muscle coordination would certainly mean death for foragers. Strong upregulation of *l(2)efl* might be a *Cataglyphis*-specific adaptation promoting worker survival.

Among heat-shock proteins, the Hsp90 family was represented by two transcripts that matched up with the Gp93 and Hsp83 protein. Heat-inducibility of *hsp83* was previously confirmed by qPCR in two species of *Cataglyphis*, including the silver ant^14^. Members of the Hsp90 family act as molecular chaperones, but they also work with a wide array of co-chaperones to regulate various biological pathways^33^. Accordingly, we found 6 transcripts directly coding for potential Hsp90 co-factors involved in signal transduction, and more specifically with the cell-cycle division (CDC37: 3 transcripts^34^, and CHORDC1: 3 transcripts^35^). Three more transcripts matched up with signal transduction proteins also involved in regulating the cell cycle: 14-3-3 zeta^36^, ANKLE2^37^, and PPP2R2A^38^. Operating with the Hsp90-CDC37 co-chaperone complex, these three proteins are involved in the Akt/PkB signaling pathway that regulates cell proliferation, survival and apoptosis^39^. This finding was bolstered by the KEGG results, which revealed enrichment in the Akt/Pkb pathway (Fig. S7). The negative impacts of heat stress on mitotic activity are well known^40^ and the HSR promotes survival by shutting down non-essential cellular processes while promoting macromolecular stability^41^. These results are consistent with a significant modulation of the cell-cycle and the ensuing rebalance of cellular resources.

As mentioned above, analysis of patterns of selection of 32 coding sequences among the 67 consistently and strongly expressed transcripts in response to heat stress indicated they were largely dominated by purifying selection. However, among four genes for which sequences were investigated for a sufficient number of other ant species, a more detailed analysis suggested that for two of them (*hsc70-4 h2* and *hsc70-5*), positive selection had occurred more often along the *Cataglyphis* lineage than along the remaining branches of the tree. Even though its coding sequence might not be under positive selection, it is possible that the specific heat-inducibility of a gene (as observed in *hsc70-5*, *hsp-60 mitochondrial*, *l(2)efl*) has evolved through modifications of its promoter region and structure of its HSE^8^. Such promoter regions may evolve quite differently in response to habitat conditions. For example, in the diptera *Stratiomys singularior*, which lives in thermally variable and chemically aggressive and hypersaline conditions, all five *hsp70* genes have different promoter regions with a unique pattern of HSE, while in the relative *Oxycera pardalina* inhabiting cold springs, all *hsp70* genes have identical promoters^42^. Given the variability of HSEs sites, determining the exact sequence and structure of *hsps* genes’ promoter regions in *C. bombycina* and comparing them to those of other related ants would be crucial to further understand the pattern of induction observed in the silver ant and the evolution of its physiological response to heat-stress.

The differential heat-inducibility of HSPs highlighted in this study could represent key adaptations to tolerate short-term and extreme thermal regime. Because triggering of the HSR is energetically costly as stress increases in frequency^43^, alternative stress-resistance mechanisms involving structural changes are likely to be selected for long-term shifts in thermal performances^44^. Examples of such mechanisms are common in extremophile organisms; they include structural transitions in thermal optimum of proteins, higher temperature threshold for triggering the HSR, or modification of biological membrane composition to adapt fluidity to novel thermal regimes^10,44–46^. Signs of increased positive selection of *hsc70-4 h2* and *hsc70-5* molecular chaperones in *Cataglyphis* may indicate that those evolutionary mechanisms for thermal resistance are at work. This premise is supported by previous studies on two northern American *Aphaenogaster* species where such structural changes, rather than an enhanced HSR, are likely responsible for the increase in upper thermal tolerance of *A. carolinensis*’s as compared to its more mesophilic relative *A. picea*^47^. However, heat-induction of *hsps* in these two species was still correlated with punctual workers acclimation to higher temperatures^18^, as is the case in *Cataglyphis*. Most ants actively adapt depth and architecture of their nest to best match their own thermal optimum^48^, and triggering the HSR might only be required when foragers exit the nest. In the Saharan silver ant both mechanisms likely co-occur to allow workers to seek food in the desert. Structural changes for long-term thermal resistance, complemented by constitutive production of Hsc70-3/Hsc70-4^13,14^ and transient production of Hsp70 co-factors, mitochondrial Hsps and small Hsps, might be the best balance between the need for a swift cellular response when foragers burst out the nest and maintenance cost of molecular chaperones. A larger scale, point-to-point comparison between heat-tolerant ant species and their mesophilic relatives would be needed to validate this scenario and unravel the evolutionary mechanisms leading to thermal scavenging in ants.

Our study highlights a specific heat-induction of several heat-shock proteins that hasn’t been reported in ant taxa so far (*hsc70-5*, *hsp60*, *hsp20*), and an increased level of positive selection in the *Cataglyphis* lineage for *hsc70-4 h2* and *hsc70-5*. This suggests that the heat-shock response of this thermal scavenger provides enhanced support to mitochondrial function and muscular tissue integrity, likely reflecting the increased need for this insect for proper motor function to face the intense stress from foraging at high speed on the burning ground. Such adaptations could give *C. bombycina* a much-needed edge in surviving the scorching heat of the Sahara Desert.

## Methods

### Field sampling and laboratory rearing

Fifteen colonies of *C. bombycina* were collected near Zagora (30°19′56″North; 5°50′18″West), in the Draa Valley of southern Morocco in early May 2015. They were reared under constant environmental conditions (25 °C, 60% relative humidity, 12:12 light-dark cycle) and fed sugar solution *ad libitum* and sliced mealworms twice a week. The colonies spent at least two months under these conditions to decrease pre-collection environmental influences before the experiment took place, which only used workers born and raised in the laboratory (*i.e*., from the egg to the adult stage). Belgium does not have ethical requirements concerning work with ants, and experiments were carried out in accordance with the relevant guidelines and regulations.

### Heat stress experiment

We used the experimental methodology described in Willot *et al*.^14^. For a given colony, 20 randomly chosen workers were selected to form 2 groups of 10 workers each separated in 50-ml glass vial containing a moist cotton ball. One group was kept at 25 °C (control treatment), and the other exposed to 45 °C (heat-stress treatment), both inside their vial submerged in a digitally controlled water bath for three hours. This procedure was replicated 4 times in 4 different colonies to obtain 4 controls and 4 heat-shocked replicates. The temperature inside the vials was monitored using 0.075-mm-diameter thermocouples connected to a digital thermometer. In *C. bombycina*, this duration of heat exposure induces a HSR without causing acute mortality^14,15^.

### RNA-seq library preparation and Illumina sequencing

The whole bodies of control and heat-stressed ants were homogenized for three minutes at maximum speed in a mixer mill using 2.8-mm zirconium oxide beads. Total RNA was extracted using TRIzol reagent in accordance with the manufacturer’s instructions. RNA was quantified with an ARN Quant-iT™ RiboGreen® Kit (ThermoFisher Scientific, CA, USA); the samples were then sent to a sequencing facility (GenoScreen, Lille, France). RNA libraries were generated using paired-end sequencing implemented by an Illumina HiSeq 2500 system in high-output mode; read length was 100 bp. After quality filtering, the mean number of reads per sample was 21.97 M (range: 18.40–25.08 M).

### De novo transcriptome assembly, transcript mapping, and identification of heat-inducible genes

To generate the reference assembly, the sequenced reads for all the samples were first combined and then assembled, using the Trinotate annotation suite (*i.e*., Trinity software; trinityrnaseq 2.2.0). Subsequently, the reads for each sample were independently mapped back onto this reference assembly, and all the transcripts were quantified using RSEM (RSEM v1.3.0)^49^. To determine which transcripts were differentially expressed in control versus heat-stressed ants, expression levels were quantified using edgeR (edgeR 3.18.1)^50^.

The edgeR model was constructed using a single pairwise comparison between two groups (HS vs NHS). The dispersion was estimated using the qCML method (estimateDisp). Differential expression between the two groups was performed using the quasi-likelihood (QL) method and a QL F-test (glmQLFit, glmQLFTest). Transcripts with a greater than background level of expression (mean log CPM > 0) and a low false discovery rate (FDR < 0.05) were used in the downstream analysis below.

### Gene ontology, functional annotation, and KEGG annotation

To understand the biological significance of the genes displaying heat-induced expression, we investigated (i) gene ontology (GO) (*i.e*., detailed annotations of gene function, related biological processes, and gene product cell locations) and (ii) Kyoto Encyclopedia of Genes and Genomes (KEGG) pathway maps (*i.e*., annotations of gene metabolic and cellular functions).

First, the transcripts were searched against NCBI’s non-redundant (nr) protein database using BLASTX. The search was restricted to arthropods and employed an e-value cut-off of 10e^−5^. Transcripts were annotated with GO terms using BLAST2GO program^51^ and an e-value cut-off of 10e^−5^. A second layer of GO terms was added to the transcripts using InterProScan online^52^, and WEGO software^53^ was used to functionally classify the terms. Second, the transcripts were annotated for biochemical pathways^54^ using the KEGG Automatic Annotation Server (KAAS) for ortholog assignment and pathway mapping^55^.

### DNA sequence variation analyses to detect patterns of selection

Among all isolated heat-induced transcripts, we retained 32 sequences for which we could identify an orthologous copy in the annotated genome of the closely related ant *Camponotus floridanus*. We isolated the coding sequence (CDS) of each one of these transcripts and conducted for each of them a classic *d*_N_/*d*_S_ test^56^ on the alignment of the sequences from both species, using the program codeml (package PAML version 4.8^57^). This test has the ability to highlight an overall pattern of negative or positive selection for a protein coding gene, by identifying a deficit or excess of non-synonymous mutations compared to expectations under a hypothesis of neutral evolution^58^. In addition, for 4 transcripts for which we found orthologous sequences in multiple ant species (see Supplementary Material for a list of species used), we conducted a likelihood ratio test to detect positive selection^59^, also using codeml. In these cases, we compared two codon-substitution models, one that assumes a single *d*_N_/*d*_S_ ratio across the entire ant phylogenetic tree, with another that assumes two different *d*_N_/*d*_S_ ratios: one for the branch leading to the genus *Cataglyphis* and another for all other branches of the tree. Another version of the two-ratio model was also created by assuming the same ratio for the branch leading to the genus *Cataglyphis* and for all branches within this genus. A likelihood ratio test was conducted to determine whether the lineage leading to Cataglyphis, possibly along with the branches within the Cataglyphis clade, is (are) characterized by a larger *d*_N_/*d*_S_ ratio than the remaining lineages of the tree. The likelihood of the ant phylogenetic tree^60^ (we used a simplified tree that included only the species for which sequences were included in the analysis, see Table S2) was computed under both the one-ratio and two-ratio models, and the two values were compared. We tested whether the two-ratio model fitted the data significantly better than the one-ratio model by comparing twice the log likelihood difference with a χ^2^ distribution (df = 1).

### Data availability

The raw transcriptomic data analyzed during the current study have been submitted to NCBI’s sequence read archive (https://www.ncbi.nlm.nih.gov/bioproject/419094) under accession no. PRJNA419094.

## Electronic supplementary material

Supplementary Information
