# Supplementary material for: Molecular chaperoning helps safeguarding mitochondrial integrity and motor functions in the Sahara silver ant Cataglyphis bombycina
Source: Sci Rep. 2018 Jun 15;8:9220. doi: 10.1038/s41598-018-27628-2 (PMC6003908; doi:10.1038/s41598-018-27628-2)
Supplement: Supplementary file 1 — Supplementary Information [file 41598_2018_27628_MOESM1_ESM.docx]

**Supplementary Information**

**Molecular chaperoning helps safeguarding mitochondrial integrity and motor functions in the Sahara silver ant *Cataglyphis bombycina***

Quentin Willot^a,^*, Patrick Mardulyn^a^, Matthieu Defrance^b^, Cyril Gueydan^c^, and Serge Aron^a^

^a^Evolutionary Biology and Ecology, Université Libre de Bruxelles, Belgium

^b^Interuniversity Institute of Bioinformatics in Brussels, Université Libre de Bruxelles, Belgium
^c^Molecular biology of the gene, Université Libre de Bruxelles, Belgium

*Author for correspondence (Quentin.Willot@ulb.ac.be)

| **Gene** | **dN/dS** |
| --- | --- |
| *14-3-3 Zeta* | 0.001 |
| *ankyrin* | 0.218 |
| *asparagine synthetase* | 0.0893 |
| *BAG2* | 0.111 |
| *calcium binding mt carrier* | 0.0942 |
| *calcyclin binding protein* | 0.0647 |
| *Chromodomain helicase DNA binding protein* | 0.05 |
| *Caseinolytic Peptidase B* | 0.1112 |
| *cystein-rich protein 2-binding protein 1* | 0.1457 |
| *cystein histidine-rich domain-containing protein* | 0.1489 |
| *dnaJ homolog subfamily A member 1* | 0.0211 |
| *endoplasmic reticulum metallopeptidase 1* | 0.1616 |
| *endoplasmin* | 0.0787 |
| *eukaryotic translation initiation factor 1A* | 0.00726 |
| *exonuclease mut-7 homolog* | 0.1443 |
| *Hsc70-3* | 0.0256 |
| *nitric oxide synthase-interacting protein* | 0.0467 |
| *nodal modulator 1* | 0.1272 |
| *phospholipase DDHD1* | 0.0747 |
| *protein phosphatase PP2A* | 0.0147 |
| *RNA polymerase gld-2* | 0.2885 |
| *SZT2* | 0.0937 |
| *transmembrane protein 120* | 0.0678 |
| *two pore calcium channel protein 1* | 0.0857 |
| *muscle M-line assembly protein unc-89* | 0.0989 |
| *uncharacterized LOC105258422* | 0.037 |
| *WD repeat-containing protein 89* | 0.1553 |

**Table S1.** *d*_N_/*d*_S_ ratios calculated for 28 CDS among the 67 consistently heat-induced transcripts between *C. bombycina* and the closely related ant *Camponotus floridanus*.

| **Gene** | ***hsc70-4 h1*** | | ***hsc70-4 h2*** | | ***hsc70-5*** | | ***hsp83*** | |
| --- | --- | --- | --- | --- | --- | --- | --- | --- |
|  | *d*_N_/*d*_S_ | Ln likelihood | *d*_N_/*d*_S_ | Ln likelihood | *d*_N_/*d*_S_ | Ln likelihood | *d*_N_/*d*_S_ | Ln likelihood |
|  |  |  |  |  |  |  |  |  |
| Single dN/dS (Ha) | 0.004 | -5002.49 | 0.018 | -5251.95 | 0.025 | -4561.78 | 0.014 | -7326.76 |
|  |  |  |  |  |  |  |  |  |
| *Cataglyphis* lineage (Hb) | 15.965 | -5013.64 | 0.059 | -5248.54 | 0.0491 | -4560.71 | 0.016 | -7326.75 |
| Other ant lineages | 0.004 |  | 0.016 |  | 0.024 |  | 0.014 |  |
|  |  |  |  |  |  |  |  |  |
| *Cataglyphis* lineage and clade (Hc) | 0.001 | -5001.49 | 0.036 | -5250.44 | 0.052 | -4559.67 | 0.009 | -7326.59 |
| Other ant lineages | 0.004 |  | 0.016 |  | 0.023 |  | 0.014 |  |
|  | Ratio | Significance | Ratio | Significance | Ratio | Significance | Ratio | Significance |
| Ha/Hb | L(Hb) < L (Ha) | n.s. | 6.82 | ** | 2.14 | n.s. | 0.02 | n.s. |
| Ha/Hc | 2 | n.s. | 3.02 | n.s. | 4.22 | * | 0.34 | n.s. |

**Table S2.** Likelihood ratio test evaluating whether the lineage leading to *Cataglyphis* (model B) or this lineage and the branches within the *Cataglyphis* genus (model C) are characterized by a larger dN/dS ratio than other branches in the ant tree (*:*p*<0.05, **: *p*<0.01). Species included in this test are *Acromyrmex echinatior*, *Atta cephalotes*, *Pogonomyrmex barbatus*, *Solenopsis invicta*, *Cardiocondya obscurior*, *Linepithema humile*, *Camponotus floridanus*, *Formica exsecta*, *Cataglyphis hispanica* and *Cataglyphis bombycina*.


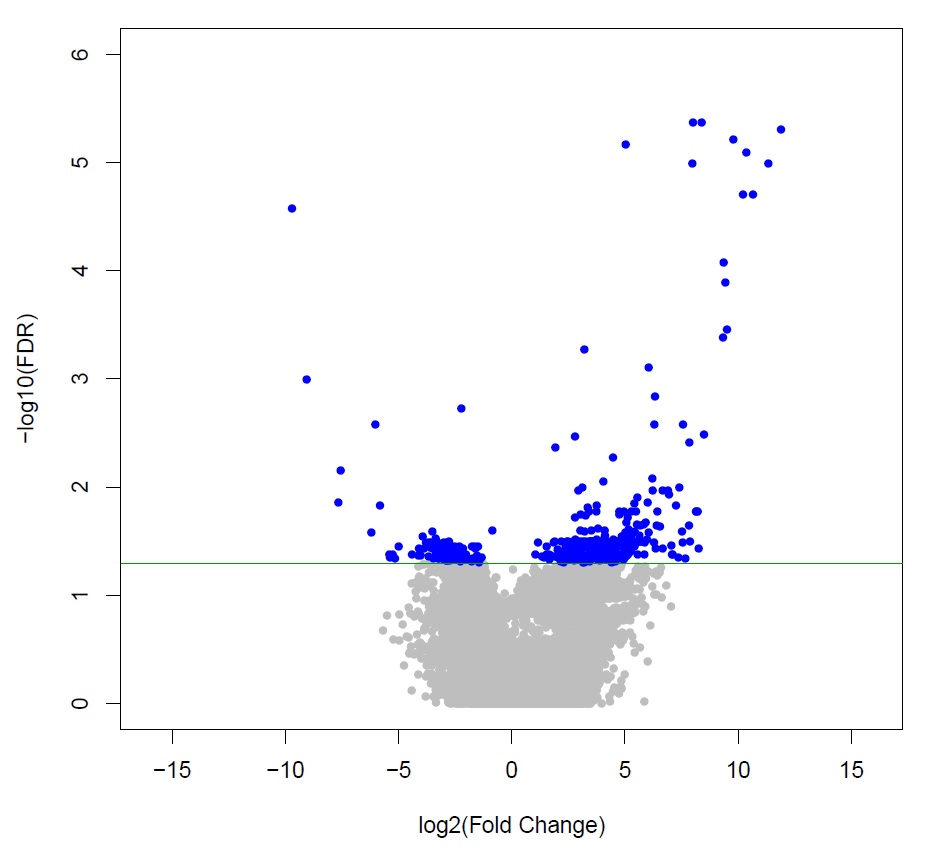


**Figure S1.** Relationship between fold change (FC) and false discovery rate (FDR) of the differentially expressed transcripts in response to heat-stress in the ant *C. bombycina*. The 533 transcripts with above background levels of expression and a FDR inferior to 0.05 (above green line) were selected for further screening.


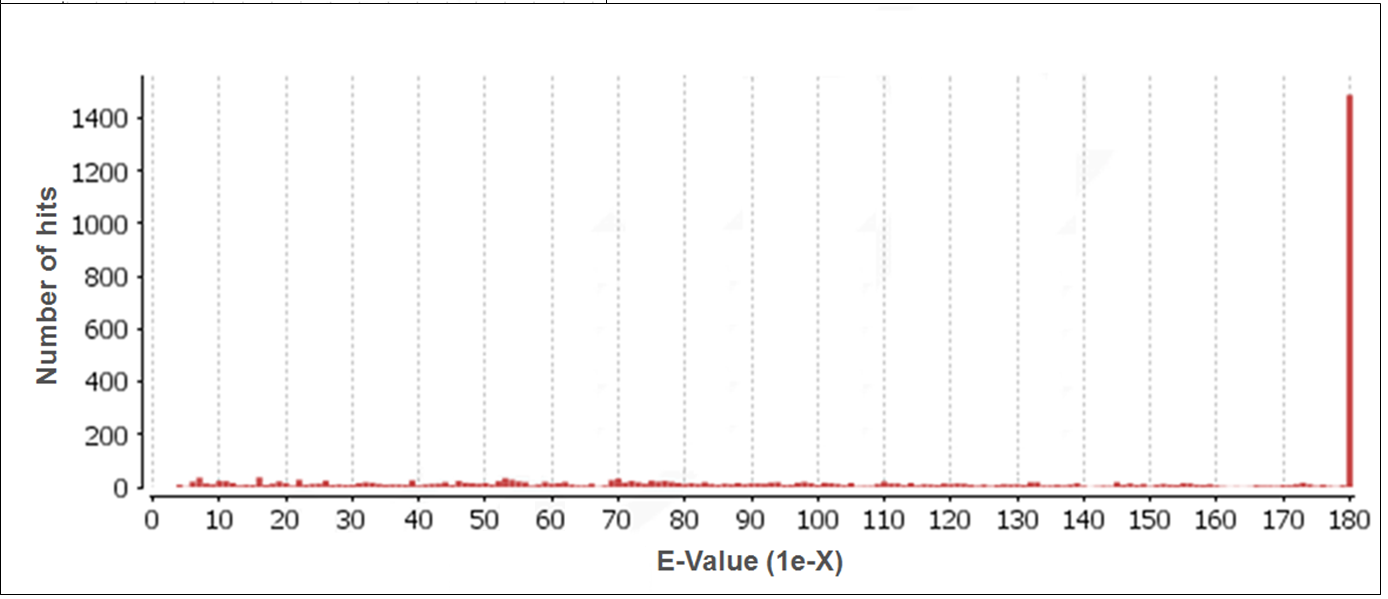


**Figure S2.** Distribution of the e-values for the 533 transcripts displaying differential expression in response to heat stress in *C. bombycina*. The e-values were obtained when the transcripts were queried against the NCBI non-redundant protein database (arthropod records only) using BLASTX. Most e-values were equal to or less than 1e^-180^, indicating transcript annotation was reliable.


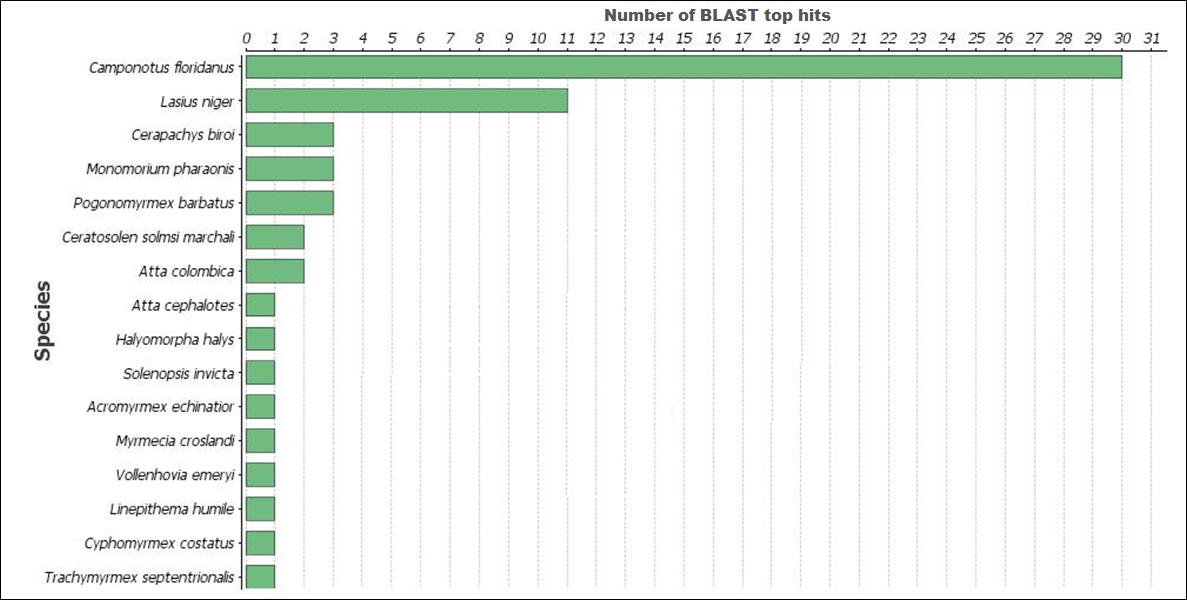


**Figure S3.** Distribution of best transcript hits with proteins from various insect species. Sixty-seven transcripts showing strong and consistent heat-induced expression (FC > 2 and RSD < 0.4) were queried against the NCBI non-redundant protein database (arthropod records only) using BLASTX (<10e^-5^). The most represented taxa for the best hit of each match were mainly other ant species.


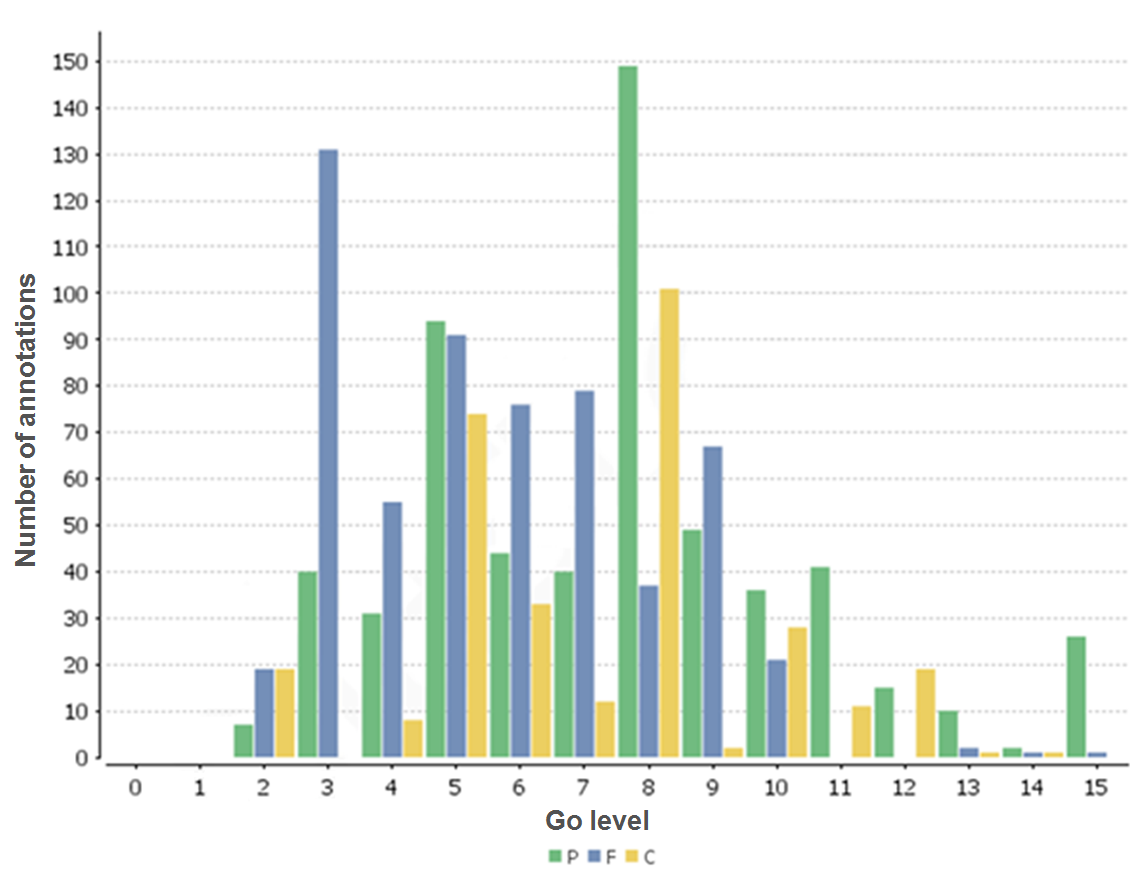
**Figure S4.** Distribution of GO levels for the 393 annotated transcripts. The transcripts were distributed across the three GO-classification domains: cellular component (GO levels 5–8), biological process (GO levels 4–8), and molecular function (GO levels 3–5). There were 1,481 annotations in total, and the mean annotation level was 6.7 (SD = 2.8).


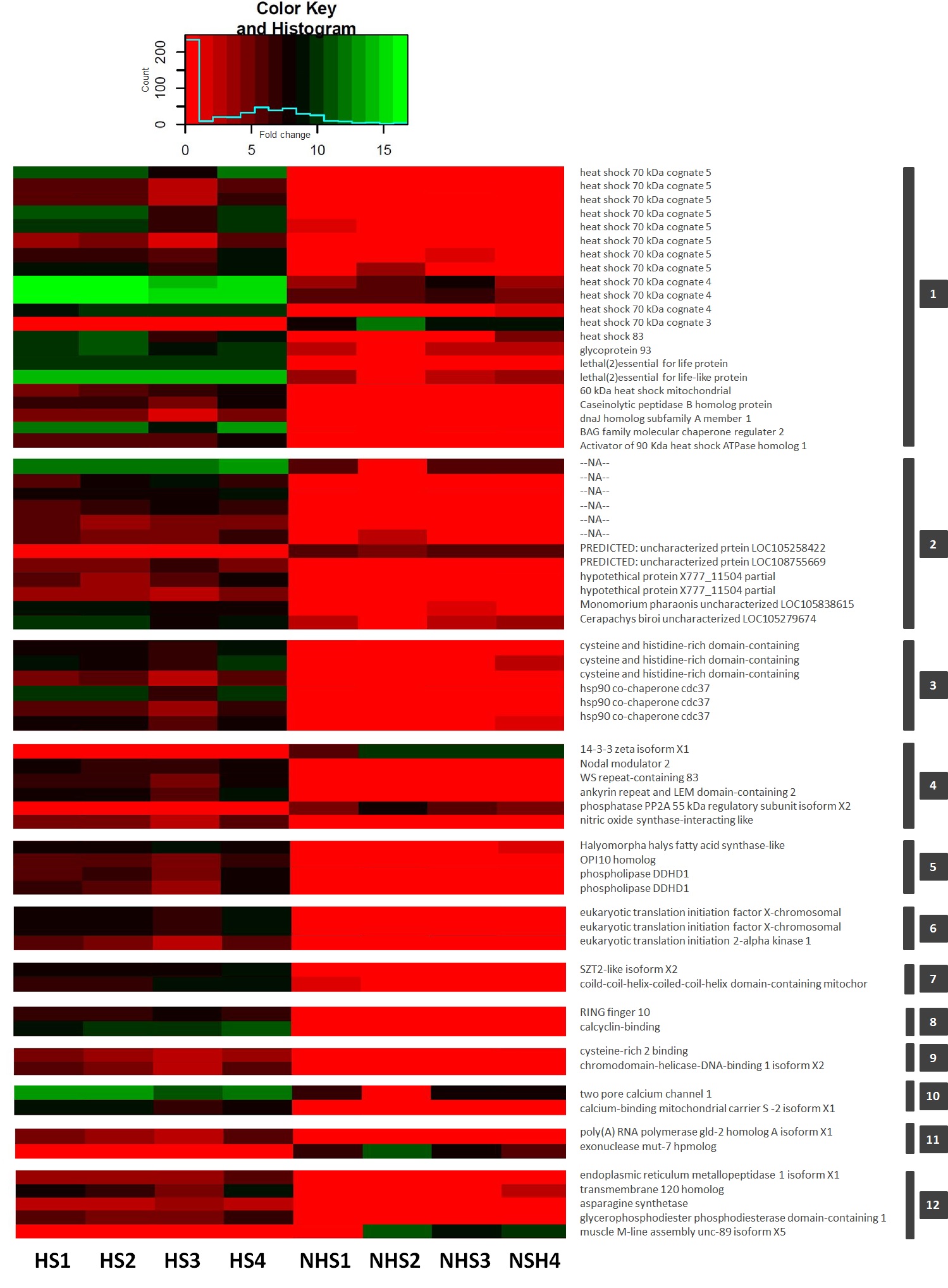
**Figure S5.** Color-coded relative expression levels and distribution curve of the 67 strongly and consistently expressed transcripts. The numeric codes signify the following: **1**: heat-shock proteins and co-chaperones, **2**: no match, **3**: Hsp90 co-chaperones, **4**: cell signal transduction proteins, **5**: lipid metabolic proteins, **6**: translation initiation factors, **7**: chromatin-remodeling proteins, **8**: proteins regulating oxidative stress, **9**: proteins in the ubiquitin‑proteasome degradation pathway, **10**: calcium transport proteins, **11**: RNA-modifying proteins, and **12**: other protein types. **HS**: heat-stress group; **NHS**: control group.


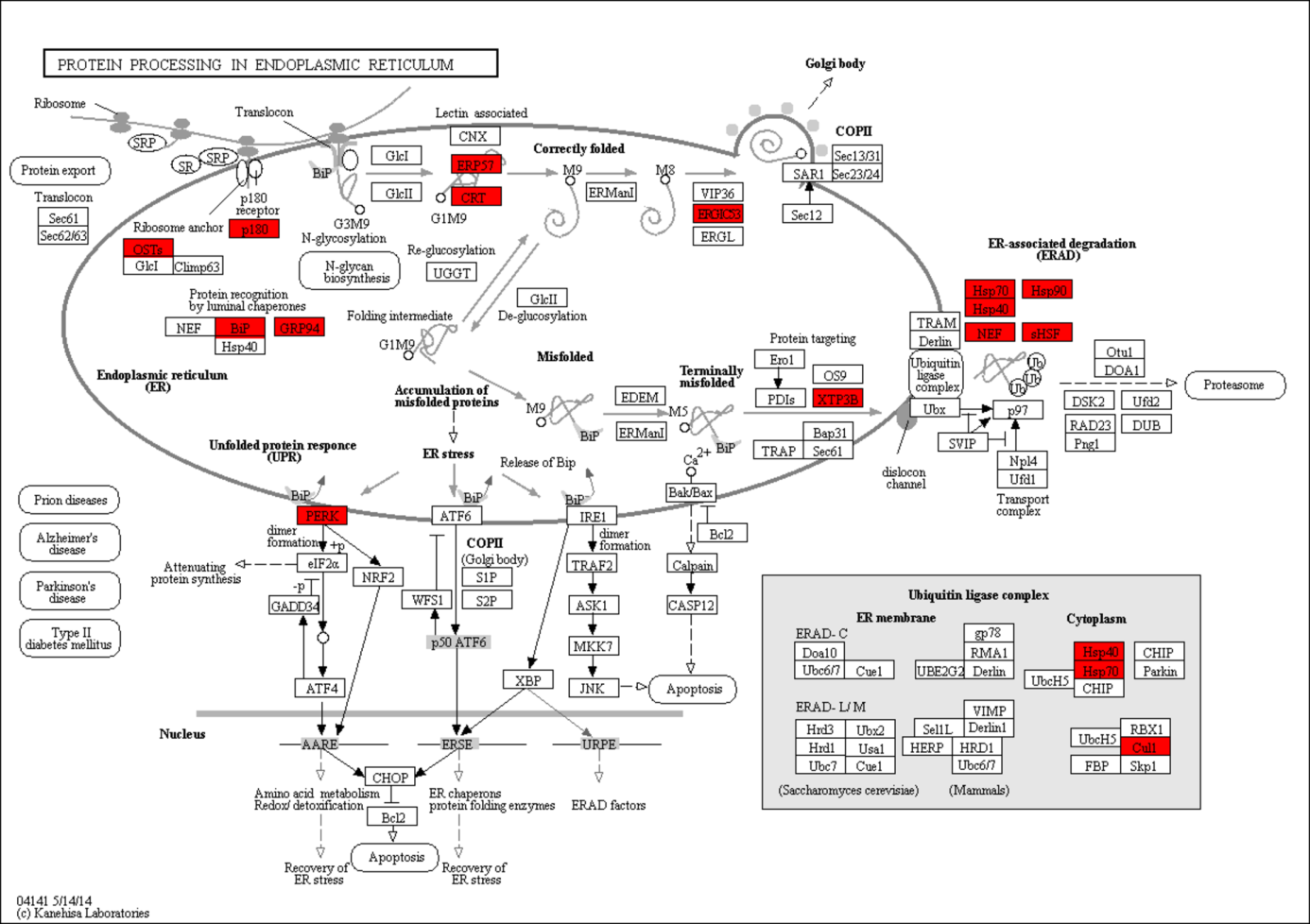
**Figure S6.** KEGG annotation pathway for transcripts involved in protein processing in the endoplasmic reticulum (map04141). The 18 positive hits are colored in red; the transcripts matched up with proteins involved in protein folding, translocation, and degradation.

**
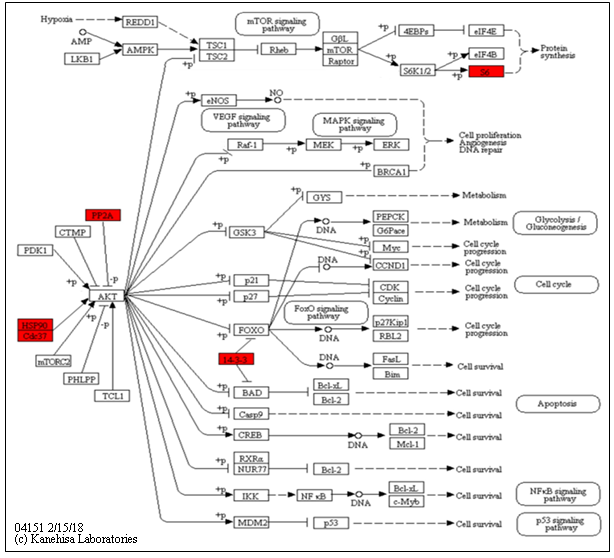
**

**Figure S7.** KEGG annotation pathway for transcripts involved in the cell cycle control via the AKT pathway (modified from map04151). Positive hits are colored in red. There is enrichment in transcripts linked with the cell cycle control and apoptosis.
